# Toward understanding the genetic bases underlying plant‐mediated “cry for help” to the microbiota

**DOI:** 10.1002/imt2.8

**Published:** 2022-03-14

**Authors:** Zhenghong Wang, Yi Song

**Affiliations:** ^1^ Institute of Plant and Food Science, Department of Biology University of Science and Technology Shenzhen China

**Keywords:** fitness, microbiota, plant, regulation, SynCom

## Abstract

Canonical plant stress biology research has focused mainly on the dynamic regulation of internal genetic pathways in stress responses. Increasingly more studies suggest that plant‐mediated timely reshaping of the microbiota could also confer benefits in responding to certain biotic and abiotic stresses. This has led to the “cry for help” hypothesis, which is supported by the identification of plant genetic regulators integrating biotic/abiotic stress signaling and microbiota sculpting. Although diverse genetic mutants have been reported to affect microbiota composition, it has been challenging to confirm the causal link between specific microbiota changes and plant phenotypic outputs (e.g., fitness benefits) due to the complexity of microbial community composition. This limits the understanding of the relevance of plant‐mediated microbiota changes. We reviewed the genetic bases of host‐mediated reshaping of beneficial microbiota in response to biotic and abiotic stresses, and summarized the practical approaches linking microbiota changes and “functional outputs” in plants. Further understanding of the key regulators and pathways governing the assembly of stress‐alleviating microbiota would benefit the design of crops that could dynamically enlist beneficial microbiota under conditions of stress.

## Highlights

- Plant‐associated microbiota affects diverse aspects of plant fitness and development, whereas how plants positively reshape a beneficial microbiota to enhance fitness is less studied.
- We reviewed the current understanding of the genetic mechanisms underlying plant‐mediated reshaping of the microbiota in response to stresses.
- Due to the complexity of the microbiota, it is challenging to confirm a causal link between microbiota changes and host phenotypes. We also reviewed the approaches to study the effect of microbiota changes on plant phenotypes.

## Graphical abstract

Diverse plant genes are positively involved in reshaping the microbiota in response to stresses, which might be a novel layer of stress tolerance strategies during evolution.

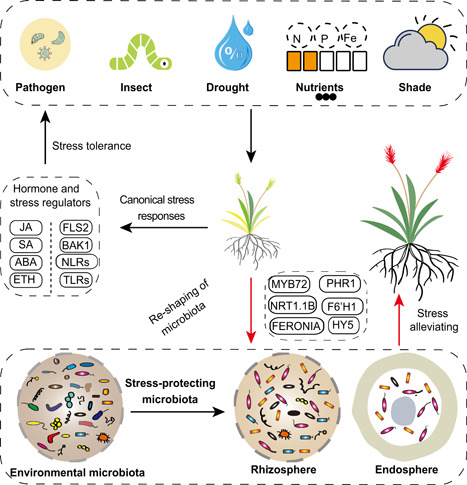

## INTRODUCTION

Global climate change, which has led to an increase in various types of stresses on agriculture, and with the increasing population worldwide have led to challenges for global food supply [1]. Application of fertilizers and pesticides boosts crop production but also generates environmental problems, and therefore, novel, environmentally friendly biotechnologies are needed to support sustainable agriculture. The host‐associated microbiome usually harbors 10–100‐fold more functional genes than the host, which could substantially boost the genetic and metabolic potential of plant–microbiota holobionts (hosts and their associated microbiota) [2]. Engineering stress‐alleviating and growth‐promoting microbiota would be an environmentally friendly way to help plants combat stresses. However, the effects of direct application of a single beneficial strain or multiple beneficial strains in the field could be unstable due to variations in local microbial communities and edaphic conditions [3, 4]. Exploration of the genetic and molecular mechanisms involved in the plant‐mediated dynamic regulation of stress‐alleviating microbiota would enable engineering of plants with improved abilities to recruit stress‐alleviating microbiota in a timely and robust manner.

**Figure 1:**
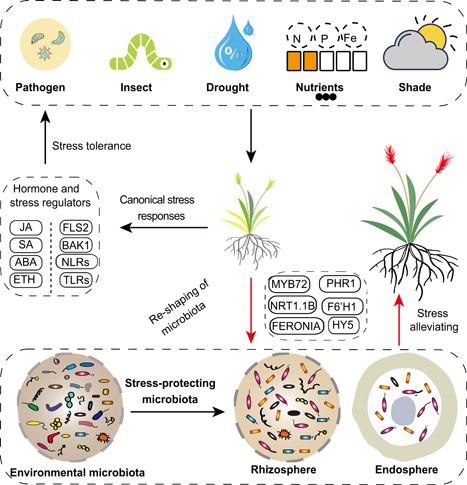
A novel layer of stress tolerance in plants: reshaping a stress‐alleviating microbiota. Plants have developed sophisticated genetic regulators to combat the biotic and abiotic stressors in terrestrial ecosystems, including pathogen infection and insect feeding, nutrient deprivation (e.g., deficient in nitrogen, phosphorus, and iron), drought, low light, or far‐red light‐enriched conditions (shade). The internal stress‐responsive pathways in plants have been widely studied (black arrows). Plants mainly utilize immune hormones (JA, jasmonic acid; SA, salicylic acid) to combat pathogen infections, and abscisic acid (ABA) and ethylene (ETH) as major abiotic stress‐responsive hormones to orchestrate stress responses. The plant membrane (FLS2, BAK1) or cytosol‐localized immune receptors (NLRs, TLRs) mediate the recognition of microbe‐related molecular patterns or pathogen effectors, which boost plant immune responses upon infection. In addition, emerging studies revealed that diverse plant genes (*MYB72, NRT1.1B, FERONIA, PHR1, F6'H1, HY5*) were positively involved in reshaping the microbiota, which might be a novel layer of stress tolerance strategies during evolution

Over the past decades, studies in plant stress biology have mainly focused on the roles of internal genetic regulators of abiotic and biotic stress responses, such as stress‐related hormones and membrane‐ or cytosol‐localized immune receptors [5, 6, 7, 8, 9] (Figure 1). The roles of the microbiota in plant stress responses are less understood. Plants can secrete 20%–40% photosynthetic fixed carbon sources into the rhizosphere and this can selectively shape a distinct root‐associated microbiota (rhizosphere, rhizoplane, and endophytic compartments) with high microbial diversity (10^3^−10^4^ OTUs [operational taxonomic units] and 10^7–8^ CFUs [colony‐forming units] per gram of rhizosphere soil) [2, 10]. The development of high‐throughput sequencing technologies has revolutionized microbial ecological research. For instance, amplicon sequencing targeting the variant region of the bacterial 16S rRNA gene or the fungal ITS gene could provide taxonomy information as well as information on the relative abundance of the microbial community [11]. Metagenomic sequencing could further provide both taxonomic and function information of microbiome [12]. Based on plant genetic mutants and microbiome sequencing analysis, it has been revealed that immune hormone (especially salicylic acid) signaling affects the microbiota composition [13], and specialized triterpene metabolites shape the species‐specific microbiota composition in *Arabidopsis* [10]. A few elaborately designed mutant screening experiments further confirmed the involvement of plant genetic factors (especially immune genes) in shaping microbiota composition [14, 15], but it still remains a challenge to confirm whether a shift in microbiota confers fitness benefits (like growth promotion or stress alleviation) in plants. We reviewed the known genetic regulators related to shaping stress‐responsive or stress‐alleviating microbiota (“cry for help”) in plants and summarized the practical approaches available to systematically confirm a causal link between a shift in the microbiota and functional outputs in plants.

## PLANT‐MEDIATED MICROBIOTA CHANGES UPON EXPOSURE TO STRESSES

### Biotic stresses enriched fluorescent *Pseudomonas* in the rhizosphere and the potential regulator FERONIA

Since the early 19th century, researchers have found that soil microbes have the potential to antagonize plant pathogens [16]. For example, pathogen infection and monoculture of wheat can generate “disease‐suppressive soil” that inhibits disease in future generations [17]. Pasteurization could inhibit soil suppressiveness, and the suppressiveness can be transferred by introducing 0.1%–10% of a suppressive soil into conductive soil [17]. This leads to the hypothesis that pathogen infection triggers host signals to recruit beneficial microbes (the “cry for help” hypothesis) in the rhizosphere. *Pseudomonas* spp. have long been found to be enriched in several disease‐suppressive soil systems and confer disease suppression through multiple mechanisms [18]. Microbiome sequencing confirmed the drastic shifting of the microbiota upon infection, and *Pseudomonas* spp. were routinely enriched. For instance, *Fusarium* wilt infection affects the composition, function, and co‐occurrence patterns of the pepper‐associated microbiome, and results in the enrichment of *Pseudomonas*, *Streptomyces*, and *Bacillus* [19]. Proteobacteria (including *Pseudomonas*), Firmicutes, and Actinobacteria are associated with disease suppression in sugar beet after *Rhizoctonia solani* infection [20]. A metagenomics study further reported that the Pseudomonadaceae, Chitinophagaceae, and Flavobacteriaceae families are enriched in the root endophytic microbiome after *R. solani* infection [21]. Interestingly, aboveground insect (whitefly) infection could also trigger changes in the root‐associated microbiota, with the recruitment of fluorescent pseudomonads in the rhizosphere [22]. Nematode infection in roots also leads to the enrichment of *Pseudomonas* spp. and disease‐suppressive soils [23]. Enriched *Pseudomonas* in the disease‐suppressive soils can directly or indirectly confer disease suppressiveness. For example, they are strong colonizers of plant roots and thus directly compete with pathogens for colonization as well as nutrients in the rhizosphere [24]. In addition, secondary metabolites such as phenazine and 2,4‐diacetylphloroglucinol from *Pseudomonas* can antagonize fungal growth directly [25, 26].

A few studies have provided insight into the mechanisms of *Pseudomonas* colonization in the rhizosphere. A genetic screen characterized a versatile receptor‐like kinase *FERONIA* (*FER*), which negatively regulates *Pseudomonas* colonization in the rhizosphere by regulating the small guanosine triphosphatase (GTPase) ROP2 and maintaining basal reactive oxygen species (ROS) levels in roots [27]. Conspicuously, the ROP pathway also regulates the soybean–rhizobium symbiosis [28], indicating a crucial role of the GEF‐ROP system in plant–commensal interactions during evolution. The role of ROS in regulating *Pseudomonas* colonization was supported by the evidence that *Pseudomonas* mutants defective in catalase (*katB*, detoxifying H_2_O_2_) activity also show defects in rhizosphere fitness [29], and catalase application to the rhizosphere could enhance the fitness of a *Pseudomonas* strain [30]. Interestingly, both the fungal pathogen *Furasium* and some nematodes can secrete RALF‐like peptides (ligand peptide of *FER*) to enhance virulence [31, 32, 33], suggesting a link between RALF production in pathogens and *FER*‐mediated regulation of *Pseudomonas* colonization. This evidence provides a plausible explanation for the origin of disease‐suppressive soils: plants can sense pathogen‐secreted RALFs (likely together with other infection/damage signals) and then recruit *Pseudomonas*. Indeed, RALF23 treatment enriches rhizosphere *Pseudomonas* in *Arabidopsis* [27], but the extent to which this pathway mediates the disease‐induced “cry for help” to enrich *Pseudomonas* remains to be studied in crops grown in the field.

### Signaling integrators between nutrient starvation and the regulation of microbiota changes

Soil nutrient content or availability directly limits the growth, yield, and quality of crops, and several studies have identified signaling integrators in plants reshaping microbiota upon nutrient deprivation. Plant roots assimilate mainly inorganic nitrogen (nitrate and ammonium) rather than organic nitrogen, and soil microbes could affect the balance between organic and inorganic nitrogen to affect nitrogen availability. Rice (*Oryza sativa* L.) *indica* varieties naturally have higher nitrogen use efficiency than *japonica* varieties, which is partially because *indica* varieties contain more diverse root‐associated microbiota with enriched nitrogen metabolism‐related functions [34]. Importantly, a master transporter of nitrogen assimilation in rice, *NRT1.1B*, is required to shape the ammonification‐related microbiome [34] (Figure 1). Metagenomic sequencing showed that the *nrt1.1b* mutant harbors a microbiota that is less abundant in ammonification process‐related functions. This provides genetic evidence that during long‐term evolution, *NRT1.1B* is required to selectively shape a beneficial microbiome that facilitates nitrogen assimilation. In maize, transcriptome and microbiome analyses in roots indicated a correlation between the expression of flavonoid biosynthesis‐related genes (including *flavone synthase type* I2, *FNSI2*) and the relative abundance of Oxalobacteraceae. This leads to the finding  that maize can synthesize flavones to enrich the rhizosphere Oxalobacteraceae and promote growth upon nitrogen starvation [35].

Although iron is abundant in soil, its availability to plants is low due to its low solubility in neutral and alkaline soils. Plants developed sophisticated iron starvation‐responsive pathways, including acidification of the rhizosphere to increase the solubility of ferric iron, secretion of a suite of secondary metabolites like coumarins (including scopoletin, fraxetin, and sideretin) to facilitate iron assimilation [36], and coordinated regulation of the microbiota. MYB72 is a master regulator of iron starvation‐induced biosynthesis of coumarins, and mutants defective in both MYB72 and Feruloyl‐CoA 6‐hydroxylase1 (F6'H1, synthetic enzyme for scopoletin) significantly shift microbiota composition upon iron starvation in limed soil (Table 1) [37]. This might due to the fact that scopoletin could selectively inhibit fungal pathogens growth, while it has little effect on growth‐promoting *Pseudomonas* [37]. Utilizing a 22‐member synthetic microbiota in a controlled system, another study confirmed that *f6'h1* mutants affect microbiota composition upon iron starvation in the rhizosphere, and found that coumarins could trigger various kinds of ROS stresses to exert antimicrobial activity [30]. Those studies confirmed that plants can secrete antimicrobial coumarins to reshape microbiota upon iron starvation, but how the shift in microbiota alleviates iron stress is unclear. A recent study showed that inoculation of SynCom (115 strains from the rhizosphere of *Arabidopsis*) could increase the iron content and fresh weight in the wild type, but not in certain coumarin biosynthesis mutants under low‐iron conditions. This indicated that coumarin‐elicited microbiota changes rather than the original microbiota are sufficient for alleviating low‐iron stress [38]. Meanwhile, transcriptome analysis revealed that SynCom inoculation relieves iron deficiency responses under low‐iron conditions, and mutants blocking iron uptake also block SynCom‐mediated rescue of the iron‐limiting stress [38]. These studies suggested that coumarin‐elicited microbiota changes enhance iron availability directly to alleviate low‐iron stress rather than boosting iron‐assimilating pathways in roots. Collectively, these studies characterized the pathway reshaping microbiota to alleviate iron starvation in detail and provide a research paradigm for systematically studying “cry for help” mechanisms in response to nutrient starvation.

**Table 1:** Plant‐mediated reshaping of microbiota composition in response to stresses

Stress	Regulator/genes/key factors	Host	Signals molecules/pathways shaping the microbiota	Effect on the microbiota	Effect of shifted microbiota on the host	References
Not tested	*FERONIA*	*Arabidopsis*	Reactive oxygen species (ROS)	Inhibiting fluorescent Pseudomonad colonization	Growth promotion and disease suppressiveness	[27]
Nitrogen deficiency	*NRT1.1B*	*Oryza sativa*	Unknown nitrate‐responsive pathways	Maintain the abundance of microbes with ammonification functions	Enhancing nitrogen‐use efficiency	[34]
Nitrogen deficiency	*FNSI2*	*Zea mays*	Flavone	Enriching Oxalobacteraceae	Growth promotion under nitrogen starvation	[35]
Iron starvation	*MYB72*	*Arabidopsis*	Antimicrobial coumarin (scopoletin)	Selectively inhibiting the soil‐borne fungal pathogens, but not the growth‐promoting *Pseudomonas* strains, inducing ROS stress in some strains	Not tested	[37]
Iron starvation	*F6'H1*	*Arabidopsis*	Coumarin (not related to the antimicrobial activity)	Shifted microbiota composition and stimulated the ability of microbiota to alleviate low‐iron stress in *Arabidopsis*	Promoting iron uptake, alleviating iron starvation responses in plants	[38]
Phosphate starvation	*PHR1*	*Arabidopsis*	PHR1 suppressed expression of immune genes	Shifted microbiota composition	Enhanced phosphate starvation responses	[39]
Phosphate starvation	*PHR1*, *MYBs*	*Arabidopsis*	Indole glucosinolate production	Avoid *Colletotrichum tofieldiae* overgrowth	Symbiosis and growth promotion	[40]
Phosphate starvation	*PHRs*	*Oryza sativa*	PHRs promoted expression of symbiotic genes	Promoted arbuscular mycorrhizal symbiosis	Promoted indirect phosphate uptake via mycorrhizal symbiosis	[41]
Dark (or weak light)	*GmSTFs*, *GmFTs*	*Glycine max*	Light‐induced translocation of GmSTF3/4 and FLOWERING LOCUS T (GmFTs) from shoots to roots	Inhibited nodulation by rhizobia	Decreased nodulation under weak light to save the energy cost of symbiosis	[42]
Suboptimal light (shade)	*MYC2*, *CRYs*, *BRI*	*Arabidopsis*	JA biosynthesis/signaling, brassinosteroid signal transduction, and cryptochromes	Shifted microbiota composition, enriched multiple *Pseudomonas* strains	Enhanced growth and immunity under shade	[43]
Drought	Unclear	*Sorghumbicoo*	Glycerol‐3‐phosphate (G3P)	Monoderm bacteria (especially Actinobacteria)	Root growth promotion under drought	[44]
Drought	Unclear	*Oryza sativa*	Unclear	Actinobacteria (*Streptomyces*)	Root growth promotion	[45]

There is a large amount of total phosphorus present in soil, but plants can only absorb orthophosphate [46]. In contrast to the positive effect of the bacterial on iron assimilation in roots, bacteria could compete with plants for orthophosphate and decrease orthophosphate content in shoots [39]. PHR1 acts as a master regulator of phosphate starvation responses and coordinates the interaction with root microbiota upon phosphate starvation. Mutants of a suite of phosphate starvation regulators or phosphate transporters shift the rhizosphere microbiota (Table 1) [39]. The association with fungal mutualists is also regulated by phosphate starvation signals. For instance, *Arabidopsis* could establish beneficial symbiosis with a growth‐promoting endophyte fungus *Colletotrichum tofieldiae* (which helps transfer phosphate to plants) only under phosphate starvation [40]. This is due to the fact that *C. tofieldiae* colonization is gated by phosphate starvation‐related production of indole glucosinolates, which could prevent *C. tofieldiae* overgrowth and is essential for the mutualism. A few regulators of phosphate starvation (*PHR1*) or indole glucosinolate production (*MYB34, MYB51, MYB122* triple mutant) are involved in maintaining a beneficial association and their mutants can block *C. tofieldiae*‐mediated growth promotion upon phosphate starvation [40]. Enlisting mycorrhizal fungi is a high‐energy‐costing process for plants and thus must be tightly regulated [47]. The association with beneficial mycorrhizal fungi is also positively regulated by the phosphate starvation regulator PHRs in rice [41], which enables rice to actively promote symbiosis and obtain indirect phosphate uptake via mycorrhizal fungi. These studies demonstrated that the association with fungal mutualists is under tight and timely control depending on the phosphate status.

The interaction between nutrient starvation and microbiota composition indicates a more general interaction between nutrient homeostasis and the root‐associated microbiota. Indeed, mutants with enhanced or disrupted root diffusion barriers (Casparian strip and suberin deposit) affect both the ionome and microbiome [48]. Meanwhile, a large amount of root‐associated bacterial isolates induce either suberization or Casparin strip formation in roots and generally affect both root ionome profiles and fitness under various kinds of mineral nutrient stresses [48]. These studies suggest that there is a broad interaction between roots and microbiota members which affects root diffusion barriers, nutrient homeostasis, and eventually fitness in plants.

### Coordinated reshaping of belowground microbiota to adapt to light stress

Light provides essential energy for photosynthesis and acts as a critical signal orchestrating growth, defense, and interactions with the microbiota. Plants have evolved sophisticated photosensing systems to perceive both light intensity and light quality, including phytochrome systems for red light and cryptochrome (CRY) systems for blue light [49]. A recent study showed that successful nodulation after rhizobial colonization in soybean roots requires light irradiation in shoots. Blue light exerts a much stronger effect in promoting nodulation in roots compared with red light, and overexpression of blue light receptor CRYs in soybean was able to enhance nodulation [42]. This process is regulated by the light‐induced translocation of GmSTF3 (an ortholog of HY5, a master transcription factor of photomorphogenesis in *Arabidopsis*) and GmFT2a from shoots to roots. Rhizobium activates calcium/calmodulin‐dependent kinase GmCCaMK to phosphorylate GmSTF3 and promotes the interaction between GmFT2a and GmSTF3, which forms the transcriptional activation complex to promote the expression of nodulation‐related genes [42]. Since nodulation and symbiosis with rhizobia are energy‐costing processes that require enough photosynthetic fixed energy, the GmCCaMK‐GmSTF3–GmFT2 pathway allows plants to precisely tune root association with rhizobia, depending on the availability of aboveground light. It would be interesting to further test whether the GmSTF3–GmFT2 pathway broadly affects the whole microbiota composition in the rhizosphere.

In nature, unfavorable light conditions such as shade (weak light intensity or long wavelength light‐enriched condition) is a common light stress in high‐density fields that causes “shade avoidance syndrome” to inhibit growth and immunity [50, 51]. Aboveground shade (low light intensity and “end of day far‐red light treatment”) could reshape a growth‐promoting microbiota (e.g., enrichment of *Pseudomonas* spp.) [43]. Inoculation with SynCom enhanced both plant growth and resistance to pathogens in shade relative to those in gnotobiotic plants. Multiple mutants defective in photoreceptors (CRY1CRY2) or regulators of jasmonic acid, gibberellins, and brassinosteroid signaling affect shade‐induced microbiota shifting, and the difference quantified by the distance in the first PCoA (Principal Coordinates Analysis) axis is also correlated with microbiota‐mediated growth promotion in shade [43]. This strongly suggests the involvement of plant genetic pathways (light perception and hormone signaling) in altering microbiota composition under shade to induce growth promotion.

### Metabolic changes are related to the drought‐induced shift in root microbiota

Drought induces drastic and conserved shifts in the microbiota composition. For instance, microbiome profiling analysis in 18 phylogenetically distant plant species revealed conserved shifts in bacterial community composition upon drought, with an enrichment of Actinobacteria, especially strains in the genus *Streptomyces* [52]. Remarkably, the relative abundance of root endospheric *Streptomyces* was correlated with drought tolerance in different plant species [53]. Several studies found that *Streptomyces* inoculation could promote growth or yield upon drought [54, 55]. Experiments in a semi‐gnotobiotic system further confirmed the link between a drought‐enriched *Streptomyces* strain and growth promotion upon drought stress [45]. This evidence demonstrated that drought‐induced microbiome changes (especially the enrichment of *Streptomyces*) can alleviate drought stress in plants. Moreover, a beneficial fungal *Piriformospora indica* (isolated from the rhizosphere of desert plants) can enhance tolerance to several kinds of stress (including drought and salinity) in plants [56, 57, 58]. The colonization of *P. indica* will trigger the biosynthesis of antimicrobial compound camalexin in roots, while drought‐responsive hormone abscisic acid (ABA) suppresses camalexin biosynthesis and promotes colonization [59]. This indicates drought‐promoted colonization of *P. indica* to enhance fitness.

The effect of drought varies depending on the time and duration of drought stress. For instance, drought stress has a greater impact on the microbiota changes before flowering than at the postflowering stage [44], and prolonged drought can cause long‐lasting “memory” changes with enriched *Streptomyces* in root endophytic microbiota, even after rewatering [45]. The integrated analysis of multiple‐omics approaches (holo‐omics) greatly furthered our understanding of plant‐mediated microbiota changes upon drought [60, 61]. Drought induces a much stronger shift in the root‐associated microbiota than that in the bulk soil microbiome or toothpick (mimicking dead roots) microbiome [53]. Metabolite profiling shows that glycerol‐3‐phosphate (G3P) is significantly enriched in roots upon drought, and metatranscriptome data suggest that carbohydrate‐, amino acid transport‐, and metabolism‐related genes are highly expressed in the root‐associated microbiota [44]. A metagenome study assembled 55 draft metagenome‐assembled genomes (MAGs), and a comparison of drought‐enriched and nonenriched Actinobacteria MAGs showed the enrichment of iron transport‐ and metabolism‐related functions in drought‐enriched MAGs [62]. This indicates that the iron starvation response in plants is correlated with increased Actinobacteria upon drought. Consistently, a maize *tom1* mutant defective in phytosiderophore transportation dampens iron uptake and also shows enrichment of Actinobacteria even without drought [62], and the relative abundance of Streptomycetaceae is significantly high in the *f6'h1* mutant (a mutant blocks the biosynthesis of iron mobilization‐related coumarins and dampens fitness in low‐iron soil) [38]. These studies confirmed the involvement of plant effects (especially low‐iron responses) in drought‐induced microbiota changes.

## APPROACHES TO LINK PLANT‐MEDIATED MICROBIOTA CHANGES AND THE EFFECT ON PLANT PHENOTYPES

### Correlation between microbe abundance and plant phenotypes

**Figure 2:**
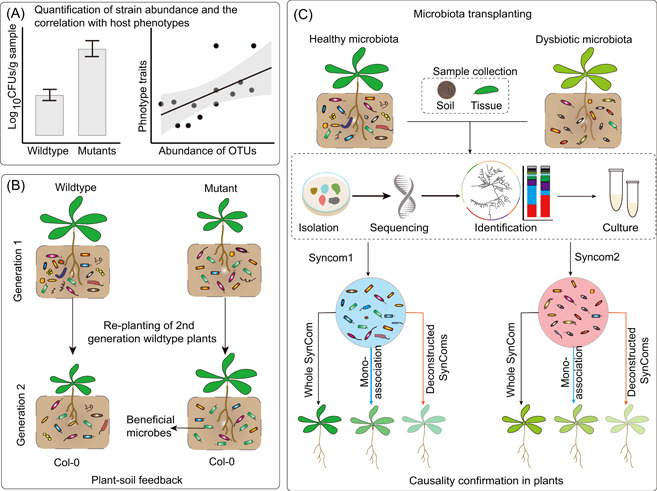
Approaches to link microbiota changes and casual phenotypes in plants. (A) For mutants affecting *Pseudomonas* or other microbes that are easy to isolate and quantify, we can quantify the CFUs directly in different groups. If the abundance of strains of interest is correlated with the plant traits (fresh weight or other stress‐related measurements), this can provide primary clues about the linkage between microbial changes and plant phenotypes. For most bacterial strains that cannot be isolated and characterized easily, we can analyze the correlation based on the relative abundance from microbiome sequencing data or PCR quantification of species‐specific marker genes. (B) A plant–soil feedback system can be used to confirm whether a changed microbiota would lead to specific phenotypes (in the diagram: growth promotion) in plants. Green bacteria indicate beneficial and growth‐promoting bacteria, which were enriched by the first‐generation plants. (C) The SynCom‐based microbiota transplantation assay could be used to confirm the causal phenotype of microbiota changes. The diagram shows that a dysbiosis causes pale and sick phenotypes in plants. To confirm the causal link, we can isolate strains from both the healthy and dysbiotic communities and perform whole‐SynCom transplantation to determine whether transplantation of the dysbiotic microbiota phenocopies the original plants with a dysbiotic microbiota. The SynCom‐based approach also provides powerful flexibility for performing either single‐strain (mono‐association) screening or combining strain modules of interest. CFU, colony‐forming units; PCR, polymerase chain reaction

We can first quantify the mathematical correlation between the abundance of specific taxa (either culture‐dependent or culture‐independent quantification) and the host traits of interest (Figure 2A). For instance, the relative abundance of endophytic *Streptomyces* after drought is significantly correlated with drought tolerance in diverse plant species [53]. This indicates that enrichment of *Streptomyces* helps plants combat drought. The levels of beneficial *Pseudomonas fluorescens* in the rhizosphere can be quantified directly by counting fluorescent colonies on King's B media (a medium that used to detect *Pseudomonas* strains). This has been used to verify a significant correlation between rhizosphere *P. fluorescens* levels and plant growth‐promoting effects [27]. Although this approach reveals only a correlation effect, it could provide clues for further testing the link using additional approaches discussed below.

### Plant–soil feedback (PSF) system

Plants can selectively shape a distinct microbiome compared with the bulk soil, and thus, first‐generation plants can shift the original soil microbiome and maintain persistent microbial “legacy” for future plants [63]. This is called PSF [64, 65], which can be used to check whether shifting of a microbiota induces certain phenotypes in the next generation (Figure 2B). A previous study reported that downy mildew pathogen infection in first‐generation *Arabidopsis* can enrich disease‐suppressive microbes in the rhizosphere soil, which could enhance disease resistance and growth of second‐generation plants in the same soil [66]. However, as the PSF effect in future generations could be due to changes in soil nutrients, two more approaches are thus needed to further confirm the involvement of microbiota changes in PSF: (1) it is essential to ensure that there are enough nutrients for the first generation and next generations (not suitable for nutrient starvation studies). Sufficient fertilizers (Hoagland or MS liquid media) must be used for both generations. (2) Pasteurization or autoclaving could be applied; if this dampens the PSF effect, it would suggest that the effect is caused by microbes rather than nutrients.

### Utilizing a SynCom to link microbiota changes and plant phenotypes

The SynCom provides a robust and controlled system to link microbiota changes and host performance (Figure 2C). Typically, 30–200 isolated microbes are selected to assemble an artificial SynCom (with a taxonomical composition similar to that of the natural soil communities) [67]. A previous study identified an immunecompromised *Arabidopsis* mutant with a dysbiotic leaf endophytic microbiota and necrosis phenotype [68]. A 52‐member bacterial community from the dysbiotic mutant could phenocopy necrosis and stunting phenotypes when inoculated on wild‐type plants growing in a gnotobiotic environment [68]. This confirmed that a dysbiotic microbiota causes the necrosis phenotype. Importantly, a collection of SynCom strains would allow researchers to flexibly select strains of interest for functional tests. A recent study clustered rhizosphere strains into four modules based on their co‐occurrence patterns in response to different environmental perturbations. By testing the effects of deconstructed SynComs (knocking out different modules and key strains) on root growth, the study further characterized a single bacterial strain (*Variovorax*) that can antagonize the root growth inhibition induced by diverse strains [69]. This highlights the power of reductionist SynCom deconstruction in revealing the effect of microbiota on host performance.

### Mathematical predication of key strains related to community functions

It is always difficult to design a perfect strain combination to best mimic the function of the original microbiome when designing a SynCom for transplantation. Recently developed machine learning methods have been effectively used to predict the disease occurrence rates in different soil communities and the maturity of the rice rhizosphere microbiome based on the composition of mathematically identified key strains [70]. This indicates the possibility of mathematically predicting the key strains (OTUs) in a community related to the functions of interest [71]. For instance, by categorizing mutants based on whether they can block microbiota‐induced growth promotion under shade, a group identified 37 strains (by a strain vector machine classifier) sufficient to predict this phenotype in the root microbiota of different mutants [43]. This guided the construction of a SynCom without those 37 strains and further confirmed their roles in growth promotion in shade, which links shade‐induced microbiota changes and plant growth promotion.

## CONCLUSION

The initial “cry for help” hypothesis originates from the observation that monoculture of crops and disease can induce microbiota changes to produce disease‐suppressive soils. However, increasingly more studies have identified genetic components involved in reshaping the microbiota to conquer abiotic stresses (especially nutrient starvation and unfavorable light conditions), indicating that this “cry for help” mechanism also exists in response to abiotic stresses. Plants can temporally (dynamically shape microbiota only under stresses) and spatially (coordinate aboveground light stress and belowground microbiota changes) control microbiota composition in response to environmental perturbations. This highlights the importance of the precise regulation of the microbiota in environment adaptation, and a single inoculation of “beneficial microbes” might not be powerful enough to combat different stresses in the field. Further understanding of the genetic and biochemical mechanisms governing the interactions between host and microbiota (especially under unfavorable conditions) would pave the way toward designing crops with stress‐inducible microbiota traits.

## CONFLICTS OF INTEREST

The authors declare no conflicts of interest.

## AUTHOR CONTRIBUTIONS

Yi Song and Zhenghong Wang conceived the idea, Yi Song and Zhenghong Wang wrote the manuscript, and Zhenghong Wang drew the figures.

## Data Availability

No new data and scripts were used for this review. Supplementary information (graphical abstract, slides, videos, Chinese translated version, and update materials) is available online DOI or http://www.imeta.science/.
